# Implementation of HILIC-UV technique for the determination of moxifloxacin and fluconazole in raw materials and pharmaceutical eye gel

**DOI:** 10.1038/s41598-022-17064-8

**Published:** 2022-08-04

**Authors:** Eman Yosrey, Heba Elmansi, Zeinab A. Sheribah, Mohammed El-Sayed Metwally

**Affiliations:** Department of Pharmaceutical Analytical Chemistry, Faculty of Pharmacy, Mansoura University, Mansoura, 35516 Egypt

**Keywords:** Chemistry, Analytical chemistry

## Abstract

Hydrophilic interaction liquid chromatography (HILIC) has inherent merits over RP-HPLC in the analyzing of hydrophilic substances. Accordingly, an innovative HILIC-UV methodology is proposed for the simultaneous estimation of ethyl paraben (PRN), fluconazole (FLZ) and moxifloxacin hydrochloride (MOX) in raw materials and pharmaceutical eye gel. The separation process was conducted using Waters XBridge™ HILIC column (100 mm × 4.6 mm, 3.5 μm particle size) at room temperature. Isocratic mobile phase containing acetonitrile: 0.1% triethylamine buffer (90:10, v/v, pH 5.0), was pumped at flow rate 1.0 mL/min and detected at 260 nm. Under these optimized conditions, PRN, FLZ and MOX showed rectilinear relationships with the concentration ranges (0.5–6.0), (5.0–50.0) and (5.0–60.0) μg/mL, respectively. The developed method offered at least fivefold increase in sensitivity within shorter time than the reported methods. Three greenness assessment tools namely: Analytical eco-scale, GAPI and AGREE were exploited to investigate the method's impact on the environment and conduct a comparative study with the reported methods. International council of Harmonization (ICH) guidelines have been followed to calculate validation parameters. The statistical comparison between results of the suggested method and the comparison method showed no discrepancy confirming accuracy of the method.

## Introduction

Microbial keratitis is a common complication of ocular surface infections in which layers of the cornea are inflamed due to bacterial, viral or fungal invasion to the underlying layers through the spaces that occurred in the corneal surface. This leads to tissue necrosis, corneal inflammation and eventually destruction. It was reported that fungal keratitis can be more pernicious and destructive compared to that caused by bacteria. Fluoroquinolones are described as first-line therapy for keratitis; in addition to anti-fungal medications in suspicion of fungal invasion^1,2^. Topical eye gel combining fluconazole and moxifloxacin hydrochloride is preferred as a single dosage form in a such case^3^.

**Figure 1:**
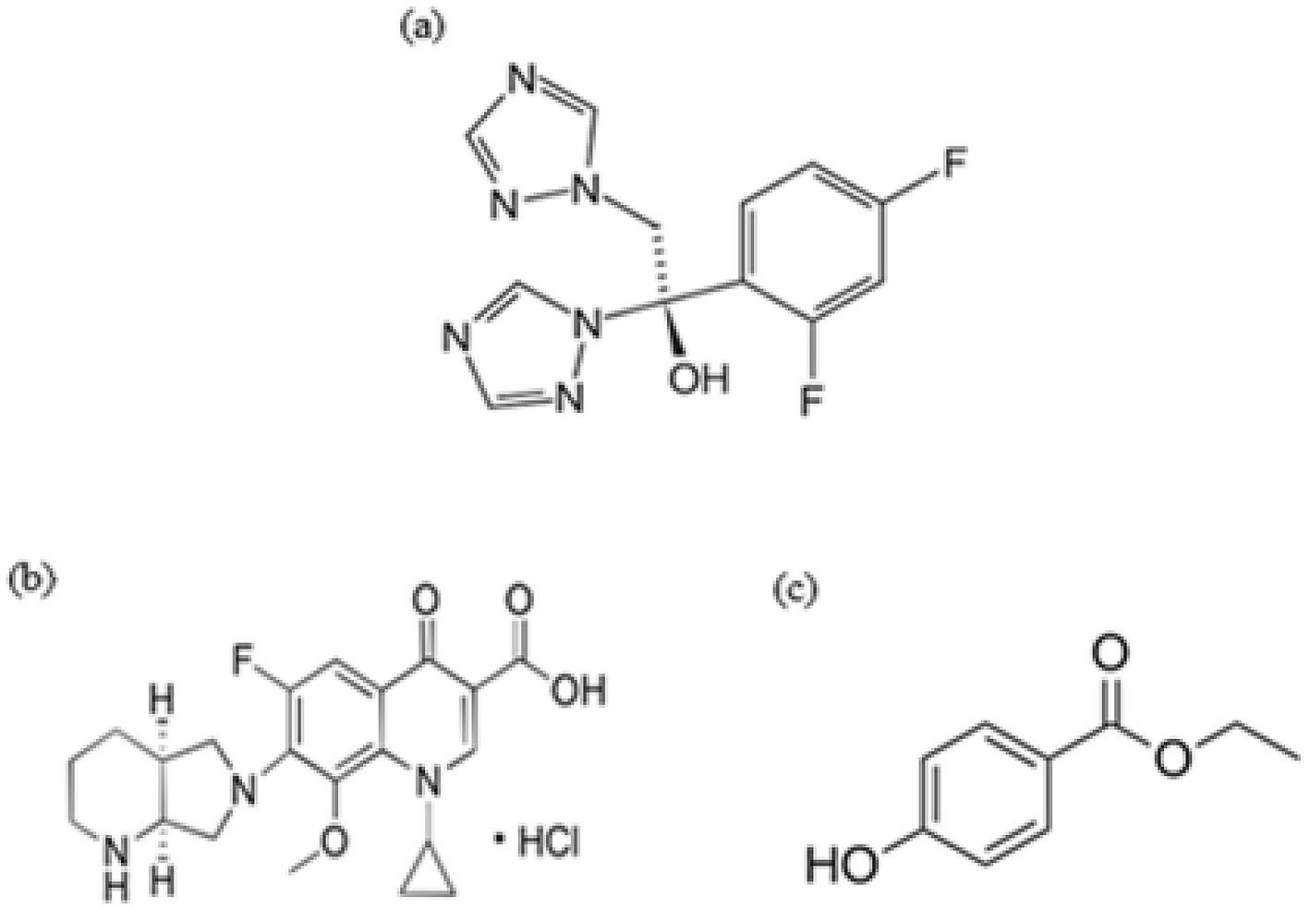
Structural formulae of: (**a**) FLZ. (**b**) MOX. (**c**) PRN.

Fluconazole (FLZ) (Fig. 1a) chemically named as 2-(2,4-difluorophenyl)-1,3-bis(1H-1,2,4-triazol-1-yl) propan-2-ol^4^, is an antifungal with a triazole ring. It is described in treating fungal infections by stopping cell growth through inhibiting ergosterol synthesis in fungal cell membrane^5^.It is used to treat candidiasis, particularly vaginal, oropharyngeal and esophageal candidiasis. Also, it is used as a prophylactic therapy against candidiasis infection in bone marrow transplantation^6^.

Literature survey revealed different methods in FLZ determination including but not limited to: spectroflourimetry^7^, spectrophotometry^8,9^, HPLC^10,11^ and voltammetry^12^.

Moxifloxacin hydrochloride (MOX) (Fig. 1b) is chemically named as 1-cyclopropyl-6-fluoro-8-methoxy-7-[(4aS,7aS)-octahydro-6H-pyrrolo[3,4-b]pyridin-6-yl]-4-oxo-1,4dihydroquinoline-3-carboxylic acid hydrochloride^4^. It is a broad spectrum fluoroquinolone with anti-bacterial activity. Its pharmacological action arises from inhibiting enzymes responsible for bacterial DNA synthesis and replication^13^. It has an excellent activity against gram negative bacteria in ocular infections^14^. It is described in treating acquired pneumonia, acute bacterial sinusitis and bacterial keratitis^2,15^

Variable methodologies have been described for determining MOX including: spectroflourimetry^16,17^, spectrophotometry^18^, HPLC^19,20^ and voltammetry^21^.

The assayed eye gel contains ethyl paraben (PRN) (Fig. 1c) as a preservative. It is chemically named as ethyl 4-hydroxybenzoate^4^.

To the best of our knowledge, two HPLC methods have been published for the simultaneous estimation of the eye gel components^3,22^. But, these methods have a low sensitivity with a long-time separation process. The aim of the developed study is to suggest a novel proposal for the implementation of hydrophilic interaction liquid chromatography (HILIC) technique for the simultaneous quantification of PRN, FLZ and MOX in their raw materials and laboratory-prepared pharmaceutical eye gel. Also, to compare the performance of the proposed method with the reported methods^3,22^.

Recently, HILIC has witnessed popularity and attention in separating and estimating food components and toxic contaminants, especially polar and ionized compounds. In contrast with RP-HPLC, both stationary phase (SP) and mobile phase (MP) in HILIC are polar. Also, the elution strength of the solvents is reversed, water is the strongest solvent in HILIC mode. SP in HILIC has been classified into different classes according to the charge of the functional groups of SP. This classification includes neutral, charged and zwitterion charged types. Ideal MP composition should contain at least 3% water and at least 60% organic solvent mostly acetonitrile (ACN). This high ratio of ACN lessened the overall viscosity of the used MP, which in turn decreases the operating pressure, allowing applying higher flow rate. In HILIC, elution order is related to the hydrophilicity of the analyte, the most hydrophilic component is the last eluted. Separation mechanisms suggested for HILIC are complex and contribute to various degrees. These include: partitioning, adsorption, ion exchange (IE) and hydrophobic retention. The separation mechanism can count on several factors concerned with physicochemical properties of SP, hydro-organic nature of MP and the structure of the assayed components^23,24^. Thus, a critical concern was introduced to optimize the separation conditions and to increase the selectivity of the SP toward the studied drugs.

Novelty of the suggested method originates from applying HILIC technique for the determination of the cited drugs which were only studied utilizing the traditional chromatographic techniques. It excelled over the reported methods in accomplishing the separation process in much less time and having a relatively high sensitivity with reasonable peak shapes. Applying the HILIC method allows overcoming the encountered limitations in the reported RP-HPLC methods.

## Experimental

### Apparatus

- Shimadzu Prominence HPLC system (Shimadzu Corp., Kyoto, Japan) with an LC-20 AD pump and SPD-20A UV–Vis detector was used.
- Waters XBridge™ HILIC column (100 mm × 4.6 mm, 3.5 μm particle size) (Ireland) was utilized to perform the proposed study. Mobile phase was degassed using Merck L-7612 solvent degasser.
- Adjusting pH through the work was performed by Consort NV P-901 pH Meter (Belgium).
- 0.45 μm membrane filter (Millipore, Ireland) was utilized to filter MP.

### Materials and reagents

- FLZ was kindly obtained from Amoun Pharmaceutical Co. El-Obour City, Cairo, Egypt.
- MOX was kindly provided by Al-Andalous Medical company, Second 6th of October, Giza, Egypt.
- PRN and sodium chloride (analytical grade) were purchased from El-Nasr Pharmaceutical Chemicals Company, Egypt.
- Poloxamer 407 (used as gelling agent), organic solvents (HPLC grade) and triethylamine (greater than or equal to 99.5%) were purchased from Sigma-Aldrich, Germany.
- Orthophosphoric acid (85%, w/v) was acquired from RiedeldeHäen, Seelze, Germany.

### Standard solutions

Stock standard solutions of FLZ and MOX were prepared separately as 200.0 μg/mL and as 100.0 μg/mL for PRN. The solutions were prepared in ACN for both FLZ and PRN and in methanol for MOX. Working solution of 50.0 μg/mL were prepared for PRN by appropriate dilution. Completing the stock solutions was aimed to be the same components of MP (ACN), but due to the lower solubility of MOX in ACN^4^, methanol was used instead. The solutions were all stable upon storing at 4 °C for 7 days.

### Chromatographic conditions used during the separation procedure

Waters XBridge™ HILIC column (100 mm × 4.6 mm, 3.5 μm particle size) was used throughout the work. The wavelength was adjusted at 260 nm using a flow rate of 1.0 mL/min. MP composition was ACN: 0.1% triethylamine buffer (TEA) in ratio of (90:10, v/v), respectively. The pH was adjusted at 5.0 using 0.2 M orthophosphoric acid then filtered using a 0.45 μm membrane filter. The separation was performed at room temperature.

### General procedure

#### Procedures for calibration curves

Different aliquots from stock standard solutions of FLZ and MOX and from working solution of PRN were separately transferred into 3 sets of 10 mL volumetric flasks covering concentration range of (5.0–50.0), (5.0–60.0) and (0.5–6.0) μg/mL for FLZ, MOX and PRN, respectively. The flasks were completed to the final mark with the MP. Under the optimum chromatographic conditions, 20 µl of each were injected as triplicates. The average peak area was plotted *versus* the corresponding concentration to get the concentration curves and to compute the corresponding regression equations.

#### Analysis of lab-synthetic mixtures

Into a series of 10 mL volumetric flasks, different aliquots of FLZ and MOX stock standard solutions and PRN working solution were transferred keeping the pharmaceutical ratio of (1:1:0.1), respectively^3^. The procedure for calibration curves was then adopted and the % recoveries were calculated utilizing the corresponding regression equations.

#### Analysis of laboratory-prepared co-formulated gel

The eye gel contains PRN, FLZ and MOX in a ratio of (0.1:1:1)^3^, so this ratio was followed to prepare the laboratory formulation by mixing 1.0, 10.0 and 10.0 mg for PRN, FLZ and MOX, respectively with the gel additives which were 2.4 gm sodium chloride and 15.5 gm poloxamer 407 all in 100 mL volumetric flask using ACN as a solvent. MOX was firstly dissolved in about 5.0 mL of methanol then mixed with other components. The flask was sonicated for 45 min and filtered using double Whatman® filter paper. Different concentrations were analyzed and the corresponding regression equations were utilized to compute percentage recoveries.

## Results and discussion

**Figure 2:**
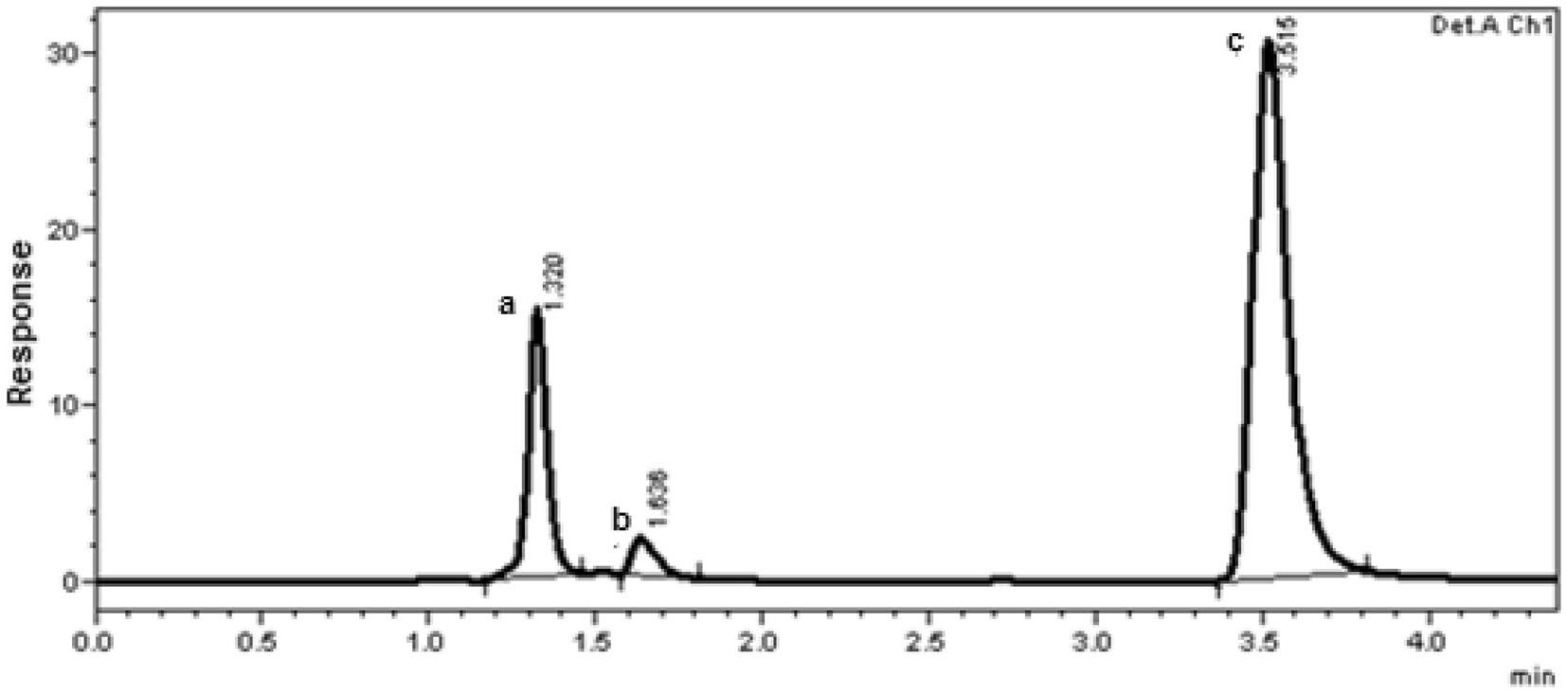
Typical chromatogram of laboratory prepared mixture of the studied components under the described chromatographic conditions. (**a**) PRN (0.5 μg/mL), (**b**) FLZ (5.0 μg/mL) and (**c**) MOX (5.0 μg/mL).

HILIC is a novel variant for RP-HPLC technique in analyzing polar compounds^23^. Comprising high percent of the organic solvent (ACN) in MP decreases its viscosity and paves HILIC to be a technique of choice for separation of hydrophilic and polar components^24^. The chromatographic conditions of the developed HILIC method were investigated and optimized to obtain the best performance in a reasonable time. The order of separation of the studied components was found to be PRN, FLZ and MOX, respectively as shown in Fig. 2. Table 1 shows the optimum chromatographic parameters obtained for the separation of the studied drugs by the proposed method.

**Table 1:** The optimum chromatographic parameters obtained for separation of the studied drugs by the proposed HILIC method.

Compound	Number of theoretical plates (NTP)	Tailing factor (T_*f*_)	Resolution (R_S_)
PRN	2079.883	1.341	
FLZ	2289.228	1.575	(PRN/FLZ) = 2.529
MOX	4818.775	1.493	(FLZ/MOX) = 11.102

### Optimization of critical parameters

#### Choice of appropriate wavelength

**Figure 3:**
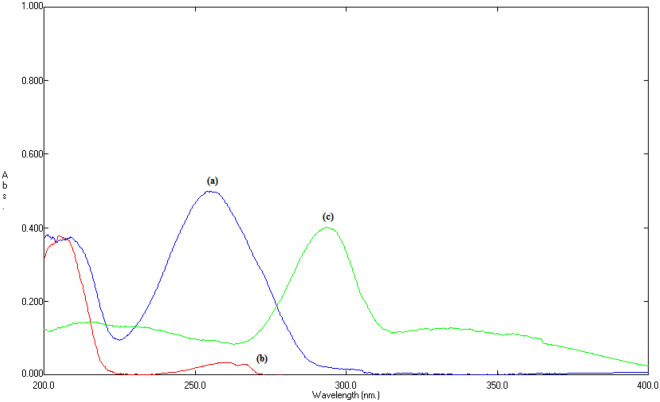
UV spectrum of: (**a**) PRN (5.0 μg/mL), (**b**) FLZ (20.0 μg/mL) and (**c**) MOX (5.0 μg/mL).

Upon recording the UV spectrum of PRN, FLZ and MOX, it was found that PRN, FLZ, and MOX have absorption maxima at 255, 260 and 295 nm, respectively. Due to the lower sensitivity of FLZ, 260 nm was selected as the most suitable wavelength for scanning the cited components (Fig. 3).

#### Mobile phase composition

An ideal MP composition in HILIC should composed of water-miscible polar organic solvents like ACN; Methanol is rarely used as water–methanol mixture is limited to induce retention to the stationary phase^25–27^. Choice of ACN is hypothesized to its miscibility with water and absence of hydrogen bond forming groups, diminishing its competition with water to solvate the surface of SP. Various modifications have been tried to achieve the best results for the three studied drugs. By trying ACN-water, MOX peak was eluted with high tailing. Replacing water by ammonium acetate buffer in different concentrations (5.0 and 10.0) mM, quietly decreased peak's tailing but still had a relatively high value. TEA was tried instead of ammonium acetate buffer and achieved the best result concerning MOX peak's tailing factor. Subsequently, the concentration of TEA was studied. Increasing the concentration higher than 0.1% didn't significantly improve the results, so 0.1% TEA was chosen as a suitable concentration.

#### pH of the mobile phase

Several pH values of the mobile phase (3.0–8.0) were studied, where pH 3.0 showed the least well-resolved peaks. Almost constant results were obtained in pH values between (4.0–6.0) with the best values according to tailing factor (T_*f*_), resolution (R_s_), and number of theoretical plates (NTP). pH values over than 6.0 showed high T_*f*_ for MOX peak and weren't preferred for column integrity, so pH 5.0 is chosen as a suitable pH. The results are summarized in Table S1.

#### Ratio of organic modifier

High percent of ACN in HILIC MP is required to induce a reasonable retention time^23^. Different ratios were tried starting from 80% ACN up to 97%. FLZ and PRN were eluted as one peak at 80% ACN. Weak resolution was observed at 85% ACN. Better resolution was achieved at 90%, further increasing showed no significant enhancement in chromatographic suitability parameters, so 90% ACN was selected as the most suitable ratio (Table S1).

#### Flow rate and column temperature

In this study, 0.8, 1.0 and 1.2 mL/min were investigated. Trying 0.8 mL/min increased the separation time, while 1.2 mL/min decreased R_s_ between PRN and FLZ. Accordingly, 1.0 mL/min was chosen as the best flow rate (Table S1). Different temperatures were tried including room temperature, 30 & 40 °C, increasing the temperature didn’t improve the results, so the separation was performed at room temperature.

#### Separation mechanism

Retention in HILIC is strongly related to the composition of MP. Incorporation of > 5% water in MP thickens the adsorbed aqueous layer on the SP and creates liquid-liquid partitioning system. Upon utilizing lower water percentage, the stagnant aqueous layer becomes thinner. This in turn downsides partitioning mechanism and allows direct interaction between the analyte and SP. In the suggested system, separation mechanism can be attributed to partitioning of the drugs between the stagnant aqueous layer on the ethylene bridged hybrid (BEH) SP and organic solvent-rich MP, and elution of the drugs according to their lipophilicity. Log P values for PRN, FLZ and MOX are 2.50, 1.0 and 0.01, respectively^28,29^. It explains the early elution of PRN and the late elution of MOX. Another explanation is polar surface area values which were 46.5, 81.6 and 82.1 Å^2^ for PRN, FLZ and MOX, respectively^28^. The highest polarity value for MOX explains its long retention on the polar SP and the lowest value for PRN explains its early elution.

### Method validation

The suggested method was validated according to ICH guidelines^30^.

Under the prescribed chromatographic conditions, a good linearity was obtained by plotting average peak area against concentration for PRN, FLZ and MOX within concentration range (0.5–6.0), (5.0–50.0) and (5.0–60.0) μg/mL, respectively. The performance of the chromatographic method is listed in Table 2.

**Table 2:** Analytical performance data for the determination of the studied drugs by the proposed HILIC method.

Parameter	PRN	FLZ	MOX
Concentration range (µg/mL)	0.5–6.0	5.0–50.0	5.0–60.0
LOD (µg/mL)	0.14	0.94	1.38
LOQ (µg/mL)	0.42	2.84	4.19
Correlation coefficient (r)	0.9998	0.9998	0.9998
Slope (*b*)	45,810.35	1027.48	46,485.46
Intercept (*a*)	82,811.37	7726.65	72,895.06
S_y/x_*	2480.63	373.06	22,587.12
S_a_*	1944.13	292.15	19,486.93
S_b_*	515.01	10.43	502.45
%Error*	0.48	0.64	0.50
%RSD	1.08	1.44	1.21
No. of experiments	5	5	6
Mean found (%) ± SD	99.91 ± 1.07	99.87 ± 1.44	99.90 ± 1.21

*N.B.

S_y/x_ = standard deviation of the residuals.

S_a_ = standard deviation of the intercept of regression line.

S_b_ = standard deviation of the slope of regression line.

% Error = RSD%/√ n.

Intra- and inter-day precisions were investigated by analyzing three concentration levels of each component three times within the same day and within three consecutive days, respectively. Satisfactory S.D, %RSD and %Error values expressed method's precision as shown in Table S2.

Using of the intercept of the standard deviation and slope values, limit of detection (LOD) and limit of quantification (LOQ) were calculated following ICH mathematical equations^30^.

LOD was 0.14, 0.94, and 1.38 μg/mL for PRN, FLZ and MOX, respectively, while LOQ was 0.42, 2.84 and 4.19 μg/mL for PRN, FLZ and MOX, respectively.

Three concentration levels of each component were tested in their raw materials adopting the reported method^22^. The reported method includes HPLC determination of the drugs using ODS-C_18_ column and mobile phase containing TEA: methanol at (58:42, v/v, pH 3.2), respectively, where pH was adjusted with phosphoric acid. The wavelength was selected at 260 nm using 1.0 mL/min flow rate. The results of the suggested and the reported methods were statistically compared using Student's *t-*and variance ratio F-tests. The comparison showed that the calculated *t* and F values were lower than tabulated values assuring the method is accurate (Table 3). Specificity of the method was assessed through quantifying different concentrations of the studied components in the laboratory-prepared eye gel. The acceptable obtained %recoveries  (Table 4) reflect absence of interference that may occur from the excipients in the formulation (poloxamer and NaCl). System suitability parameters were calculated to check the competence and feasibility of the developed methodology in terms of NTP, Tf and Rs. The results shown in Table 1 points out that, the values of the suggested method were in acceptance with USP criteria revealing efficiency of the evolved approach [The United States Pharmacopoeia 40 and National Formulary 35. US Pharmacopoeial Convention, Rockville. 2017].

**Table 3:** Application of the proposed method and the comparison method for the analysis of the studied drugs in raw materials.

Parameters	Proposed method			Comparison method^22^	
	Amount taken (μg/mL)	Amount found (μg/mL)	Found (%)	Amount taken (μg/mL)	Found (%)
PRN	0.5	0.498	99.60	2.0	99.50
	1.0	0.988	98.80	3.0	100.37
	3.0	3.051	101.70	5.0	99.94
	5.0	4.982	99.64		
	6.0	5.989	99.82		
X^−^ ± SD			99.91 ± 1.07		99.94 ± 0.44
Student'*s t-*test			0.04 (2.45)*		
Variance ratio (F test)			6.10 (19.25)*		
FLZ	5.0	4.895	97.90	20.0	98.23
	10.0	10.050	100.50	30.0	101.12
	20.0	20.347	101.74	50.0	99.83
	30.0	29.759	99.20		
	50.0	49.998	100.00		
X^−^ ± SD			99.87 ± 1.44		99.73 ± 1.45
Student's t-test			0.13 (2.45)*		
Variance ratio (F test)			1.02 (6.944)*		
MOX	5.0	4.935	98.70	20.0	98.02
	20.0	20.228	101.14	30.0	101.90
	30.0	30.117	100.39	50.0	99.63
	40.0	39.302	98.26		
	50.0	50.548	101.10		
	60.0	59.879	99.80		
X^−^ ± SD			99.90 ± 1.21		99.85 ± 1.95
Student's t-test			0.05 (2.36)*		
Variance ratio (F test)			2.58 (5.786)*		

*Values between parentheses are the tabulated *t* and F values^31^.

### Analysis of different matrices

The proposed method showed eligibility in the simultaneous determination of the studied components in different concentrations of their synthetic mixtures with accepted percentage recoveries, the results are shown in Table S3. Moreover, estimation of the studied components in the laboratory-prepared co-formulated gel by performing the developed methodology showed reasonable percentage recoveries. The reported method^22^ was also adopted to quantify the studied components in the laboratory prepared co-formulated gel. The results of the developed method were compared statistically with the reported one^22^ and showed no significant difference proving method's accuracy (Table 4)^31^.

**Table 4:** Assay results for the determination of the studied drugs in laboratory-prepared gel.

Parameters	Proposed method						Comparison method^22^		
	Amount taken (μg/mL)	Amount taken (μg/mL)	Amount taken (μg/mL)	Found (%)	Found (%)	Found (%)	Found (%)	Found (%)	Found (%)
PRN/FLZ/MOX	2.0	20.0	20.0	101.30	98.51	99.53	99.20	98.56	99.51
	3.0	30.0	30.0	100.68	99.46	99.33	99.50	100.09	99.20
	5.0	50.0	50.0	98.77	99.98	100.26	99.95	99.57	98.42
X^−^ ± SD				100.25 ± 1.32	99.32 ± 0.62	99.71 ± 0.49	99.55 ± 0.38	99.41 ± 0.78	99.04 ± 0.56
Student's t-test				0.88 (2.78)*	0.14 (2.78)*	1.54 (2.78)*			
Variance ratio (F test)				12.20 (19.00)*	1.09 (19.00)*	1.32 (19.00)*			

*Values between parentheses are the tabulated *t* and F values^31^.

### Greenness evaluation and comparative study

Serious concern was raised toward method's impact on the environment, especially when such large volume of ACN was used. Studying the eco-friendly profile was performed using three assessment tools to study the all insights for the method’s greenness. These tools include: Analytical eco-scale, Green Analytical Procedure Index (GAPI) and Analytical GREEnness (AGREE). Analytical eco-scale is concerned with volume and hazard of both the used solvents and the resulted waste in addition to instrument's energy. Each parameter is given penalty points and the total score is subtracted from 100 (the ideal score)^32^. GABI is a more descriptive tool which encompasses almost all principles of green analytical chemistry (GAC). A pictogram with five pentagrams, colored with green, yellow or red reflecting good, intermediate or bad impact on the environment^33^. The recent AGREE tool is an easily-used software calculator for evaluating the greenness property. It is classified into 12 sections relying on GAC principles. It provides both qualitative and quantitative data. Each section colored with either green, yellow or red reflects the ecological impact, with a total score in the middle from 0 to 1 (1 is the greenest)^34^.

**Figure 4:**
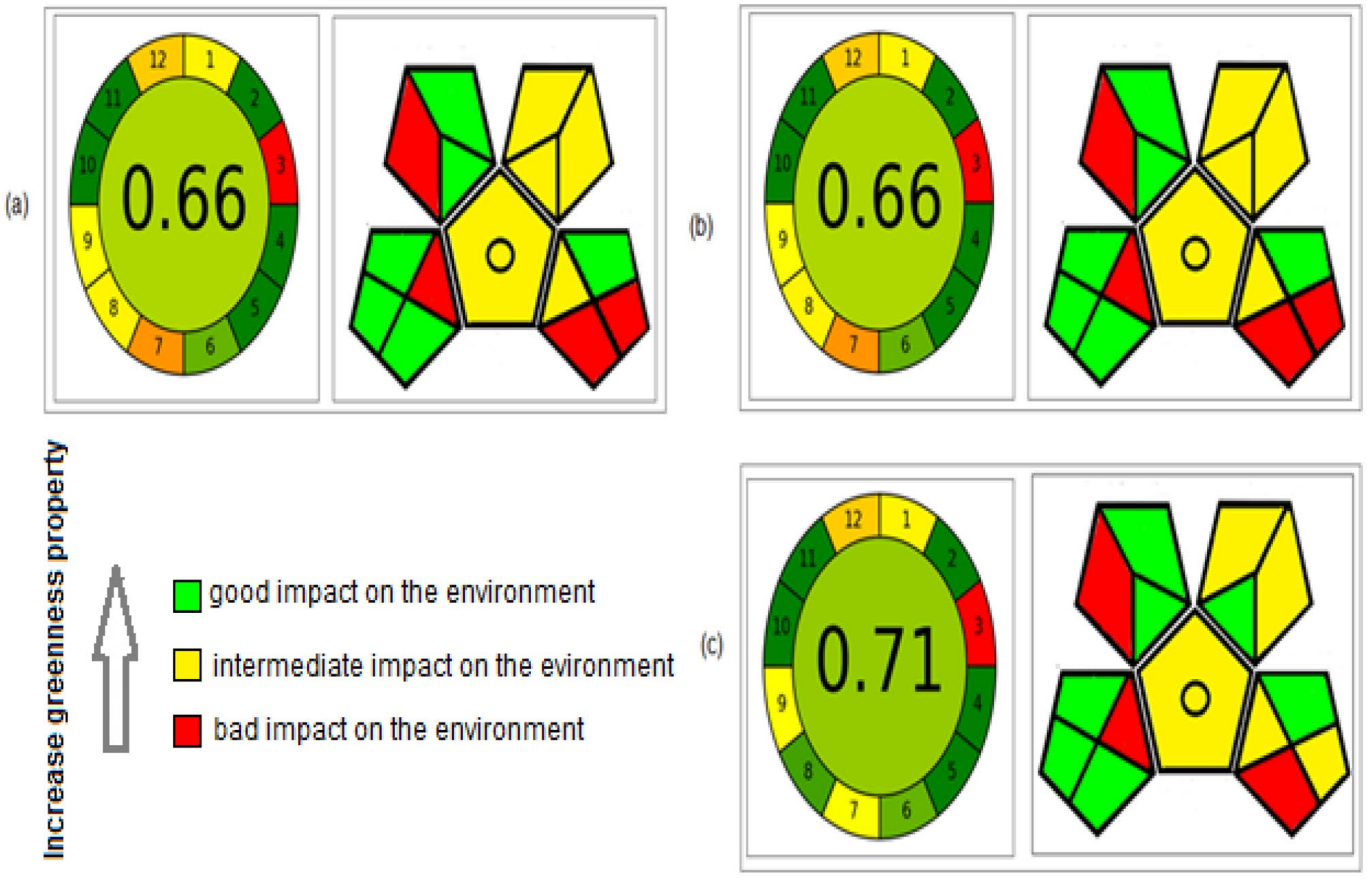
Greenness evaluation by AGREE and GABI, where (**a**) Evaluation for the reported HPLC method^3^. (**b**) Evaluation for the reported HPLC method^22^. (**c**) Evaluation for the suggested HILIC method.

A comparison of the eco-friendly property between the suggested HILIC and the reported HPLC methods^3,22^, was performed using the aforementioned tools. The greenness profile comparison shown in Table S4 and Fig. 4 elucidate superiority of the proposed HILIC method over the reported methods.

Upon comparing the results of the suggested approach with the reported methods, it was found that the suggested method offers either a fivefold or a 20-fold increase in sensitivity more than the reported methods^22^ and ^3^, respectively. Moreover, it accomplishes the separation process within four minutes, much less time than 20 min in the reported methods. This decrease in the separation time offers consumption of fewer quantities of organic solvents, adding time and cost saving properties to the developed method, also producing less waste that helped in saving the environment. The developed method provides a greenness evaluation using three different metric tools which wasn't studied in the reported methods. As a consequence, applying the HILIC method allows overcoming the encountered limitations in the reported RP-HPLC methods.

## Conclusion

Keeping up with the modern hyphenated analytical separation methods, evoked us to adopt the HILIC technique in our work. The developed methodology allowed the simulatenous estimation of PRN, FLZ and MOX in raw material and laboratory-prepared eye gel. Chromatographic conditions were optimized to get the best separation conditions. A comprehensive comparative study was performed between the suggested and the comparison methods' greenness profile using Analytical eco-scale, GAPI and AGREE assessment tools. Acceptable percentage recoveries were obtained from analyzing different concentrations of synthetic mixtures and laboratory-prepared eye gel proving method’s selectivity and specificity. In a nutshell, the proposed HILIC method's features make it a suitable candidate for quality control labs.

## Supplementary Information

Supplementary Information.

## Data Availability

The datasets generated and/or analyzed during the current study are available from the corresponding author on reasonable request (Eman Yosrey).
