# Supplementary material for: Implementation of HILIC-UV technique for the determination of moxifloxacin and fluconazole in raw materials and pharmaceutical eye gel
Source: Sci Rep. 2022 Aug 4;12:13388. doi: 10.1038/s41598-022-17064-8 (PMC9352657; doi:10.1038/s41598-022-17064-8)
Supplement: Supplementary file 1 — Supplementary Information. [file 41598_2022_17064_MOESM1_ESM.docx]

Table S1: Optimization of the chromatographic conditions for the separation of PRN, FLZ and MOX by the proposed HILIC method

Table S2: Precision data for the determination of the studied drugs by the proposed HILIC method.

Table S3: Assay results for the determination of the studied drugs in their synthetic mixtures.

Table S4: Greenness evaluation for the proposed and reported methods by analytical eco-scale.

Table S1

| **Retention time** | | | **Tailing Factor (T*_f_*)** | | | **Resolution (R_S_)** | | **Number of theoretical plates (NTP)** | | |  | |
| --- | --- | --- | --- | --- | --- | --- | --- | --- | --- | --- | --- | --- |
| **MOX** | **FLZ** | **PRN** | **MOX** | **FLZ** | **PRN** | **(FLZ/MOX)** | **(PRN/FLZ)** | **MOX** | **FLZ** | **PRN** |  |  |
| 4.034 | 1.822 | 1.342 | 1.311 | 1.906 | 1.409 | 10.754 | 2.907 | 6438.513 | 1200.533 | 2005.734 | 4.0 | **pH of mobile phase** |
| **3.959** | **1.689** | **1.331** | **1.091** | **1.756** | **1.485** | **12.542** | **2.676** | **6178.314** | **1677.394** | **2006.414** | **5.0** |  |
| 4.556 | 1.690 | 1.328 | 1.463 | 1.887 | 1.447 | 14.212 | 2.467 | 6213.389 | 1542.043 | 1919.536 | 6.0 |  |
| **3.229** | **1.618** | **1.328** | **1.493** | **1.575** | **1.341** | **11.102** | **2.529** | **4818.775** | **2289.228** | **2079.883** | **90:10** | **Ratio of organic modifier** |
| 3.959 | 1.689 | 1.331 | 1.091 | 1.756 | 1.485 | 12.542 | 2.676 | 6178.314 | 1677.394 | 2006.414 | 95:5 |  |
| 17.585 | 2.065 | 1.364 | 1.228 | 2.587 | 1.484 | 29.033 | 3.893 | 7279.653 | 1138.809 | 2232.760 | 97:3 |  |
| 7.022 | 2.187 | 1.677 | 1.432 | 2.012 | 1.398 | 16.911 | 2.922 | 6243.208 | 1635.648 | 2552.025 | 0.8 | **Effect of flow rate (mL/min)** |
| **3.229** | **1.618** | **1.328** | **1.493** | **1.575** | **1.341** | **11.102** | **2.529** | **4818.775** | **2289.228** | **2079.883** | **1.0** |  |
| 2.801 | 1.361 | 1.103 | 1.467 | 1.603 | 0.926 | 9.621 | 2.181 | 4302.408 | 1794.190 | 1674.998 | 1.2 |  |

| **PRN** | | | **FLZ** | | | **MOX** | | |
| --- | --- | --- | --- | --- | --- | --- | --- | --- |
| **Sample concentration** | **Repeatability** | **Intermediate precision** | **Sample concentration** | **Repeatability** | **Intermediate precision** | **Sample concentration** | **Repeatability** | **Intermediate precision** |
| **2.0 µg/mL** | | | **20.0 µg/mL** | | | **20.0 µg/mL** | | |
| **Mean found** (%) **X`** | 100.67 | 100.04 | **Mean found (%) X`** | 98.55 | 100.12 | **Mean found** (%) **X`** | 98.89 | 100.88 |
| **± SD** | 1.34 | 0.62 | **± SD** | 1.43 | 1.62 | **± SD** | 1.51 | 0.26 |
| **%RSD** | 1.33 | 0.62 | **%RSD** | 1.45 | 1.62 | **%RSD** | 1.53 | 0.26 |
| **%Error** | 0.77 | 0.36 | **%Error** | 0.84 | 0.94 | **%Error** | 0.87 | 0.15 |
| **3.0 µg/mL** | | | **30.0 µg/mL** | | | **30.0 µg/mL** | | |
| **Mean found** (%) **X`** | 100.14 | 101.17 | **Mean found** (%) **X`** | 100.10 | 100.47 | **Mean found** (%) **X`** | 99.92 | 100.97 |
| **± SD** | 0.55 | 0.53 | **± SD** | 1.45 | 1.27 | **± SD** | 1.69 | 0.59 |
| **%RSD** | 0.55 | 0.52 | **%RSD** | 1.45 | 1.26 | **%RSD** | 1.69 | 0.58 |
| **%Error** | 0.32 | 0.30 | **%Error** | 0.84 | 0.73 | **%Error** | 0.98 | 0.33 |
| **5.0 µg/mL** | | | **50.0 µg/mL** | | | **50.0 µg/mL** | | |
| **Mean found** (%) **X`** | 99.29 | 99.35 | **Mean found** (%) **X`** | 100.00 | 99.79 | **Mean found** (%) **X`** | 99.88 | 100.27 |
| **± SD** | 1.43 | 0.29 | **± SD** | 0.44 | 0.21 | **± SD** | 1.01 | 0.84 |
| **%RSD** | 1.44 | 0.29 | **%RSD** | 0.44 | 0.21 | **%RSD** | 1.01 | 0.84 |
| **%Error** | 0.83 | 0.17 | **%Error** | 0.25 | 0.12 | **%Error** | 0.58 | 0.48 |

Table S2

Intermediate precision (also known as ruggedness) expresses within-laboratory variation, as on different days, or with different analysts or equipment within the same laboratory.

Repeatability refers to the use of the analytical procedure within a laboratory over a short period of time using the same analyst with the same equipment.

Table S3

| **Parameter** | **Amount taken**  **(μg/mL)** | **Amount taken (μg/mL)** | **Amount**  **taken (μg/mL)** | **Found (%)** | **Found (%)** | **Found (%)** |
| --- | --- | --- | --- | --- | --- | --- |
| **PRN/FLZ/MOX** | 2.0 | 20.0 | 20.0 | 100.63 | 102.39 | 101.23 |
|  | 3.0 | 30.0 | 30.0 | 102.73 | 99.00 | 100.41 |
|  | 5.0 | 50.0 | 50.0 | 99.52 | 100.00 | 101.13 |
| **X^-^ ± SD** |  | | | 100.96  **±** 1.63 | 100.46 **±**1.74 | 100.92  **±** 0.45 |

Table S4

| **Reagents/ Technique** | **Proposed HILIC method** | **Reported HPLC method [3]** | **Reported HPLC method [19]** |
| --- | --- | --- | --- |
| **Acetonitrile**  **Trietylamine**  **Orthophosphoric acid**  **Methanol** | 2  2  1 | 2  1  4 | 2  1  4 |
| **HPLC** | 1 | 1 | 1 |
| **Waste** | 3  3 | 5  3 | 5  3 |
| **Occupational hazard** | 0 | 0 | 0 |
| **Total penalty points** | 12 | 16 | 16 |
| **Analytical eco-scale total score** | 88 | 84 | 84 |
